# Disease-focused evaluation of AI-ECG tools: clarifying study design, findings, and the principles of open science

**DOI:** 10.1093/ehjdh/ztag120

**Published:** 2026-07-27

**Authors:** Gamze Babur Guler, Arda Guler, Ibrahim Halil Tanboga

**Affiliations:** University of Health Sciences, Mehmet Akif Ersoy Thoracic and Cardiovascular Surgery Training and Research Hospital, Department of Cardiology, Istanbul, Türkiye; University of Health Sciences, Mehmet Akif Ersoy Thoracic and Cardiovascular Surgery Training and Research Hospital, Department of Cardiology, Istanbul, Türkiye; Atlas University Faculty of Medicine, Cardiology & Biostatistics, Istanbul, Türkiye

We thank Dhingra *et al*. for their thoughtful engagement with our study and, most importantly, for making these AI-ECG tools freely available to the research community.^1–5^ We agree that independent evaluations should clearly distinguish the intended use of a model, the study population, the operating thresholds, and the specific inference that a given design can support.

A central point of clarification is the scope of our study. Our cohort consisted entirely of patients with confirmed hypertrophic cardiomyopathy (HCM); therefore, it was not designed to estimate formal diagnostic discrimination between HCM and non-HCM individuals. Accordingly, we did not report area under the receiver operating characteristic curve (AUROC), sensitivity, or specificity for HCM detection against a non-HCM comparator group. As stated in our manuscript, our aim was not to replicate or revalidate the diagnostic performance reported in the original development and validation studies, but rather to conduct a post-validation, disease-focused assessment of model behaviour in a well-characterized confirmed HCM cohort.^1^

In this context, the term performance was used in a broad disease-focused sense, referring to the distribution of AI-generated outputs, their agreement with manually assigned ECG diagnostic categories, and their associations with clinical, electrocardiographic, echocardiographic, and cardiac magnetic resonance markers of disease expression. We agree that this should be kept distinct from formal diagnostic accuracy or discrimination. To avoid ambiguity, our interpretation is that the study characterizes within-HCM model behaviour rather than providing a diagnostic validation study of HCM detection.

For the same reason, our analyses should not be interpreted as conventional calibration assessment for the binary endpoint of HCM diagnosis. In a case-only cohort, the observed HCM status is fixed by design. Our emphasis was therefore on probability distributions and clinical correlates of the AI-derived scores, not on calibration of predicted probabilities against HCM incidence in a mixed population. This distinction is consistent with broader guidance that AI models may require evaluation across multiple domains, including but not limited to discrimination.^6,7^

We also appreciate the correspondents’ point regarding validated operating thresholds. The 50% and 75% values reported in our manuscript were intended as descriptive summaries of the score distribution, not as validated diagnostic cutoffs or proposed clinical operating points. We agree that operating thresholds are model specific and depend on the development population, validation framework, and intended use setting. Therefore, our main inference does not rest on these descriptive cutoffs. Rather, the full score distributions, together with the observed severity associations, provide the most informative description of model behaviour in this confirmed HCM cohort.

Case-only evaluations have a legitimate role when the question concerns disease expression, treatment response, monitoring, or biological signal within an established disease population. Such designs have been used to evaluate patient-level AI-ECG HCM scores during mavacamten therapy, across serial HCM assessments, and within other inherited arrhythmia cohorts.^5,8–10^ We agree, however, that they answer a different question from diagnostic validation. A clinically representative validation cohort including both cases and non-cases remains necessary to determine diagnostic discrimination, threshold-based classification performance, and clinical deployment characteristics in a screening or diagnostic setting.

The most important contribution of our study is therefore the characterization of clinically meaningful heterogeneity in AI-ECG outputs across the HCM disease spectrum. Hypertrophic cardiomyopathy model probabilities were associated with maximal wall thickness, N-terminal pro-brain natriuretic peptide (NT-proBNP), left atrial diameter, Sokolow–Lyon index, T-wave axis, late gadolinium enhancement, and HCM phenotype, with apical HCM showing the highest probabilities.^1^ Similarly, PRESENT-SHD probabilities were associated with NT-proBNP, QTc interval, QRS duration, T-axis, left atrial diameter, left ventricular ejection fraction (LVEF), tricuspid annular plane systolic excursion (TAPSE), late gadolinium enhancement (LGE), diffuse LGE, atrial fibrillation, and bundle branch block.^1^ These findings suggest that AI-ECG outputs may capture gradients of phenotypic expression and structural or functional disease burden, even when they are not being evaluated as binary diagnostic classifiers.

This interpretation also aligns with the correspondents’ own observation that the reported associations may indicate preservation of biologically meaningful signal in a complex disease-enriched cohort. Rather than viewing the case-only design as a substitute for diagnostic validation, we view it as a complementary approach that can reveal phenotype-specific model behaviour that may not be evident from aggregate case–control metrics. The finding that AI-derived probabilities varied across HCM phenotypes and were markedly lower in the small subgroup with normal ECGs is clinically informative for understanding how these tools behave across the spectrum of established disease.

The correspondents also raise important points regarding post-intervention patients and technical deployment. Our cohort included a small proportion of patients with prior myectomy, alcohol septal ablation, or implantable cardioverter defibrillator (ICD) implantation, reflecting real-world HCM practice. We acknowledge that prior interventions and acquisition details may influence ECG morphology and model outputs, and this was one of the reasons our conclusions were framed as disease-focused model characterization rather than diagnostic deployment guidance. Future collaborative analyses with time-stamped ECGs, stratification by treatment status, and detailed documentation of preprocessing would further strengthen interpretation.

Finally, we agree that spectrum and case-mix effects are central to AI-ECG evaluation. The solution is not a single preferred study design, but alignment between study design and research question. Diagnostic deployment requires clinically representative cohorts with realistic disease prevalence, appropriate comparators, and prespecified thresholds. Disease-focused evaluations, such as the present study, can provide complementary information about severity gradients, phenotype-specific outputs, and biological signal within a confirmed disease cohort. Both approaches are needed for responsible translation of AI-ECG tools into clinical practice.

In conclusion, our study should be interpreted as an independent, disease-focused characterization of AI-ECG outputs in a large confirmed HCM cohort. It does not estimate formal diagnostic discrimination or threshold-based diagnostic performance for HCM detection. Its principal findings are the heterogeneous probability distributions, phenotype-specific output patterns, agreement analyses, and clinically relevant associations between AI-derived scores and markers of HCM severity. We are grateful for the correspondents’ open science approach and welcome collaborative efforts that combine independent disease registries with model-specific methodological expertise.

## Data Availability

Data available on request. The data underlying this article will be shared on reasonable request to the corresponding author.
